# Dry Eye Disease: An Overview of Its Risk Factors, Diagnosis, and Prevalence by Age, Sex, and Race

**DOI:** 10.7759/cureus.54028

**Published:** 2024-02-11

**Authors:** Niyati Deo, Prachee Nagrale

**Affiliations:** 1 General Medicine, Jawaharlal Nehru Medical College, Datta Meghe Institute of Higher Education and Research, Wardha, IND; 2 Ophthalmology, Jawaharlal Nehru Medical College, Datta Meghe Institute of Higher Education and Research, Wardha, IND

**Keywords:** gender-based differences, races, dry eye disease, tear film, meibomian gland dysfunction

## Abstract

This short review focuses on the significance and prevalence of dry eye disease (DED) in the arena of ophthalmology. DED can be identified as one of the most common optical morbidities affecting about one-fourth of the patients visiting ophthalmology clinics.

The perception of the cytology and disease evolution of DED has shown a noteworthy advancement in the last decade by recognizing two diverse mechanisms of the disease: tear desertion and deficient tear production. The role of these two components independently or concurrently in the prevalence of DED was also understood. Several studies in different parts of the world have projected that DED is more common in women as compared to men and this difference increases with ageing. Aged people, especially women in the menopausal and post-menopausal stages, are more prone to DED. This ailment is more prevalent in patients suffering from autoimmune diseases with a higher percentage of women getting affected. Various everyday activities as well as social and dietary behaviors like smoking might set off DED symptoms. Extensive visual tasking while using a computer, watching television, and doing a lot of reading also increase the risk of DED.

Although DED occurs in all age groups, it is seen in very few children in comparison to adults. In fact, DED in children may be related to diverse factors such as congenial, inflammatory, and autoimmune disorders as well as environmental conditions and nutritional deficiencies. A significant relationship has also been found between DED and racial differences among individuals. A few studies have suggested that the Asian population is more susceptible to DED as compared to the Caucasian population, but this concept needs further research and investigation. Climatic conditions and environmental challenges, such as relative humidity (RH), internal atmosphere, effluence, travel by air, and intense temperatures, are equally important in the occurrence of DED.

The present review aims to examine the prevalence of DED in relation to age, sex, and race by analyzing several relevant studies and also have an overview of the diagnosis and risk factors of the disease.

## Introduction and background

Dry eye disease (DED) is one of the most widespread visual ailments recognized by eye aridness, uneasiness, and sensitivity to light. A quarter of people who visit ophthalmology clinics experience dry eye [1]. A majority of eye care professionals deal with this rapidly expanding public health issue. If DED remains untreated, it may become persistent and can have an extensive effect on the individual's quality of life [2]. Henrik SC Sjögren, a Swedish ophthalmologist, was the one who noticed the interplay of dry mouth, keratoconjunctivitis sicca, and joint pain occurring in about 90% of female patients [3]. The phrase "dry eye" was first used in 1950 by Andrew de Roetth, and for a while, it was believed that the decrease in the aqueous layer of the tear film was what caused the dryness of the eye [4].

According to medical science, there are many etiologic reasons, numerous amalgamations of entomological and pathological expressions, and diverse grades of DED severity regarding the dysfunction of tear film [5]. The International Dry Eye Workshop (DEWS), in 2007, gave a new definition to DED and also classified it into three parts based on the causes, mechanism, and severity of the disease [6]. The new definition given by the DEWS was: "Dry eye is a multifactorial disease of the tears and ocular surface that results in symptoms of discomfort, visual disturbance, and tear film instability with potential damage to the ocular surface". According to the new definition of the condition, inflammation of the ocular surface and increased tear film osmolarity are also observed [1].

DED can also be classified as sporadic or persistent. Sporadic dry eye may occasionally occur due to environmental conditions and visual tasks with reduced blinking, whereas persistent dry eye constantly undergoes symptoms and damage to the ocular surface. The main symptoms of dry eye experienced by patients are grittiness, itching, foreign body impression, tearing, burning, visual fatigue, and dryness.

Research has shown that DED occurs more with the increase in age and is more prevalent in women of older age than men. DED is more prevalent in people with comorbidities affecting about 8% of the population [7], 78% of them being women [8]. DED is also predominant in post-menopausal women [9] and older people [10,11]. The occurrence of DED ranges between 7.4 and 33.7% which depends upon several factors, such as how the disease is identified, the nature of the population that has been surveyed, and the studies that have been taken into account [11,12]. Epidemiological studies have identified a number of risk factors that accelerate the development of DED, including advancing age and female sex. It is possible that this is the case because women's tear production noticeably declines after the age of 60. A review of the etiology of DED has recently taken place, and substantial strides have been made in this area. One of these is the discovery of two distinct illness mechanisms, namely, inadequate tear production and tear desertion. Each of these mechanisms has a crucial role in the occurrence of DED, either independently or together [13].

The objective of the present study is to have an overview of the prevalence of DED and its effect on sex differences, racial differences, and various age groups. It also aims to understand the risk factors associated with DED along with diagnosis procedures.

## Review

Research methodology

The present review was carried out from April to June 2023. Various research papers and reviews were examined using PubMed and Google Scholar databases. Scopus and Web of Science databases weren't included, as more relevant literature was found in PubMed and Google Scholar. Data was extracted mostly from the papers published between 2003 and 2018. Research papers related to DED were carefully studied with a special focus on their prevalence by age, sex, and race. The relevant search terms used were dry eye disease, ocular surface, tear film, signs and symptoms, prevalence, risk factors, diagnosis, etc. It was quite a complicated task to dig out the required information from the existing studies, as DED is not yet considered a growing challenge for ophthalmologists. As a result, very limited authentic data was available about its prevalence by age, sex, and race. Looking at the rapidly growing number of DED patients globally, this area requires a lot of attention from researchers.

The effect of sex differences on DED

Like many other ocular conditions, DED disproportionately affects women. It is identified and diagnosed earlier due to the better use of the healthcare system by them. Exclusive healthcare settings designed for women mostly offer services through single windows to meet their therapeutic needs and are primarily utilized by younger women. This setting helps in providing additional opportunities to recognize, learn, instruct, and provide treatment to women suffering from DED or at peril of developing DED. The studies on DED currently do not throw enough light on the interplay between DED and women's health. This gap makes it complicated for practitioners from fields other than eye care to identify female patients at risk of DED, as this disease is multifactorial with erratic signs and symptoms reported by the patients [14,15]. Improved threat perception and faster recognition of DED symptoms and indicators may help expedite referrals to an ophthalmologist [16].

Recently, the in-depth analysis carried out by the 2017 Tear Film and Ocular Surface Society (TFOS) DEWS II about the prevalence of DED revealed that the pervasiveness of the signs and symptoms related to DED have been increasing day by day in the last few decades starting at the age of 40-49 years [17]. Women are more likely to get DED symptoms as they get older, i.e., 14% at 50 and 22% at 80 and older. Contrarily, in men, this tendency is less pronounced and manifests later, with DED symptoms rising from 7% of men aged 60-69 to 13% of men aged 80 and older [18].

Findings of the Beaver Dam Offspring Study also pointed out that the occurrence of DED in men was 10.5%, whereas in women it was found to be 17.9%. The study found that hormone medication for women increased the risk of DED [2]. To determine the rate of prevalence of DED, a comparison was done between the 2009 Physician's Health Study (PHS; number of males of 50+ years of age, n=25,000) and the 2003 Women's Health Study (WHS; number of females of 49+ years of age, n=39,000) [19]. After analysis of data, it was revealed that the patient's mean age when they were diagnosed with DED was 66 years for men and 60 years for women. Assessment using questionnaires projected that women were more commonly having severe symptoms [18]. On analysis of these studies, it was found that in the United States, the prevalence of DED in women was double (3.25 million) as compared to that of men (1.68 million) [19,20]. In 2017, the analysis done by the National Health and Wellness Survey (NHWS) data revealed that inequalities between genders in the occurrence of DED increased with age [21]. According to certain reports, the incidence of DED was higher in young women than in males in the same age group, i.e., 2.9% women vs. 2.6% men in the age group of 18-34 years. With a difference of 22.8% vs. 12.6% for respondents beyond the age of 75, this disparity significantly increased [21].

The differentiation of DED pervasiveness for men and women comes under the sphere of gender effects on well-being. This affluent area of study has recognized gender differentiation in reaction to healing agents, exploratory and curative interventions, gender disparities in surroundings related to autoimmune situations, and a variety of gender dissimilarities in the case of general ailments [22]. Variations in DED occurrence are endorsed to gender disparities, which add to the risk of DED and its appearance, resistant response, and treatment reaction that is explicit to women.

Biological sex disparities influencing optical composition, performance, and physical condition are illustrated from the molecular point of view to the physiological scrutiny level. The molecular point of view comprises differentiation in tissue morphology, gene appearance, protein amalgamation, and the functioning of the mesothelium [23]. These differences become responsible for disparities in the production of aqueous tears, lipid manufacture, mucous emission, blink rate, ocular impervious functioning, and stability of tear film, which may add to DED signs and symptoms. Sex-related disparities in optical functioning have been acknowledged in the conjunctiva, cornea, meibomian glands (MGs), and lacrimal glands, as well as other eye configurations [24]. These physiological disparities may be responsible for the divergent DED occurrence rates perceived in men and women. For instance, sex-related alterations in the cornea arise in conditions like menopause, the onset of the menstrual cycle, and pregnancy. This includes differentiation in hydration, thickness, curve, pigmentation, and sensitivity of the endothelium [22].

Research has recommended that the menstrual cycle may change the ocular exterior balance due to modifications in estrogen levels, inducing symptoms of dry eye in female patients [25]. During the follicular stage, the estrogen peak is responsible for lesser tear creation and constancy, dryness of the surface, and irritation [26]. Gender-related disparities in the inherent and adaptive impervious responses of the optical fascia are minimal if systemic diseases are not present. The close interplay of autoimmune diseases and DED unreasonably influence women. If the immune system is impaired, immunity differences on the ocular surface increase between men and women [27]. Research has proven that the risk of symptomatic DED is greater in women with thyroid disease or depression in comparison to men [16]. Observations suggest that these symptoms increase with age. Women of <65 years of age suffering from diabetes mellitus were at a higher risk of symptomatic DED, and persistent migraine, headache, and depression in the last 12 months were seen to be added risk factors for those who were ≥65 years of age [16].

There is a marked relationship between dry eye and autoimmune diseases which are more prevalent in women, such as systemic lupus erythematosus (SLE), Sjögren's syndrome, rheumatoid arthritis (RA), and thyroid diseases [28]. Rosacea, a persistent inflammatory disease, is commonly related to DED. These diseases are mostly associated with women in the majority of cases [29,30]. Patients suffering from rosacea often show ocular signs and symptoms like photophobia, distorted vision, burning, irritation, tearing, and red eyes [31]. The reason for the optical alterations in these patients may be swelling of the tissue surrounding the MGs followed by changes in the lipids produced by the glands. Tear volume is also reduced in many patients with ocular rosacea [32].

Many studies have found that there is some relationship between DED and chronic pain [33-36]. Like DED, female sex and older age are considered to be risk factors for many chronic pain syndromes [37,38]. Pain-related symptoms are much more apparent in women with DED as compared to men with the same disease. This shows the probability of sex-related underlying biological mechanisms [23]. Iatrogenic factors and cultural behavior are also responsible for increasing the risk of DED in women. Iatrogenic factors consist of plastic surgeries, the use of contact lenses, elective ophthalmic procedures like periorbital surgeries, and both universal and contemporary remedial treatments, such as the use of allergy eye drops [39-41]. Behavioral causes of DED comprise superficial periorbital surgeries and the use of tropical cosmetics and facial creams [27]. Most of these procedures are largely used by women who subsequently fall prey to DED.

Self-reports of women in many studies have projected that they lack the following indicators much more than their male counterparts [42]. Balanced nutrition, minimal stress, sufficient sleep, steady moods, anxiety, and depression are significant indicators that define overall physical and mental health [43-47]. Health-associated quality of life and apparent health are worse among females with DED as compared to their male counterparts. It has been reported by the patients of DED that tasks like television viewing, computer work, night driving, and prolonged reading become difficult for them, thus negatively affecting their quality of life [47].

Looking at the gender disparities in behaviors related to care seeking and utilization of healthcare services, it is quite evident that women interact more and in a different manner with the healthcare system. They come forward for early detection, referral, and precautionary treatment of DED. Women have been shown to explore a wider range of behaviors, such as dietary supplements and other appropriate therapies [48,49]. When compared to males, women take omega-3 fatty acids more frequently to alleviate their DED symptoms according to research by Schaumberg et al. [18]. DED can be a multifaceted and prolonged condition in terms of treatment and management. People suffering from DED experience intractable symptoms. Several studies have revealed that gender-related dissimilarities between women and men are responsible for these differences especially as they age.

Impact of DED on various age groups

Children and Adolescents

Although DED occurs in all age groups, it is least prevalent in children. There is a possibility that children may report fewer symptoms than adults for the same level of visual damage leading to underreporting and underdiagnosis of the disease [50]. The paediatric age group has a vast range of causes and treatments for DED. In reality, a variety of additional variables, including autoimmune, inflammatory, and congenial diseases, environmental factors, and nutritional inadequacies, may all contribute to DED in children [51]. The lack of literature on DED in children is a major difficulty, and the necessity to expand our knowledge in this field cannot be ignored. Currently, the majority of researchers have made an effort to learn more by examining the medical histories of young population with DED. 

Donthineni et al. performed a cross-sectional investigation on 1023 newly diagnosed DED patients who were under the age of 21 and were diagnosed with DED between 2010 and 2018. Data analysis revealed that meibomian gland dysfunction (MGD), vitamin A deficiency (VAD), and Stevens-Johnson syndrome (SJS) were the three main causes of DED, accounting for 49%, 33%, and 9% of cases, respectively. After dividing the data into different age groups, it was found that SJS and VAD were responsible for aqueous deficiency dry eye (ADDE) in 92% of infant patients, 96% of toddler patients, 76% of early childhood cases, and 68% of middle childhood cases. The main cause of DED in 51% of patients in early and 66% in late adolescence was MGD and evaporative dry eye [50]. The causes of optical impairment due to DED were found to be different in various age groups, with ADDE due to SJS being the most common among them.

Diagnosis of dry eye is often ignored because it is very uncommon in children and its nature is little known in children than in adults. Many autoimmune, inflammatory, congenital, and endocrine disorders may be responsible for this disease in children, or it may be prevalent under certain ecological and dietary conditions. If DED is detected early, it will be possible to treat the underlying cause. If the condition is not addressed on time, it may become a chronic issue that needs to be well managed to avoid ulceration and scarring of the optical surface. As dry eye is a disease associated with numerous conditions, it requires a comprehensive approach towards diagnosis and treatment [52].

Adults

DED is a degenerative condition associated with ageing. It has been projected through several studies that it is a growing public health burden throughout the world with the increase in the ageing population. The equilibrium between the optical surface and tear film, which characterizes the disease, may have a notable influence on ocular function, visual comfort, quality of life, and work productivity [53].

The pervasiveness of several optical surface disorders is seen in human beings with growing age. DED is one of the most prominent disorders among these. Scientific data on ocular surface disorders cannot be always categorized clearly due to the overlap of symptoms. When modern eye drugs with preservatives are used concurrently with chronic allergic reactions in the ocular surface in elderly patients, the condition may be misdiagnosed as dry eye [54]. Patients with dry eyes are more likely to be sensitive and experience symptoms similar to allergic rhinoconjunctivitis [55]. The strong correlation between the naturally occurring flora of the gut and the occurrence of respiratory diseases is comparable to conjunctival approval. Age has an impact on this type of immunological control in the eye, just as it does in the digestive and respiratory systems. Ageing is accompanied by an increase in immune-based ocular surface problems, which is known as age-related conjunctival tolerance mutilation [56].

According to a transverse study based on a presumptive registry database that abided by the principles of the Declaration of Helsinki, the burden of the ageing population is anticipated to rise with time. The study found that advancement in age was a major risk factor for DED, and it was projected that this problem would amplify globally with the increase in the ageing population. MGD symptoms first surfaced during the third decade of life, initially in the natural history of illness progression. The short stoppage in the life cycle before the commencement of old age should be used as a window of opportunity for searching for preventive interventions at this age [53].

Racial differences and DED

Many studies have proven that there is a significant relationship between DED and racial differences among individuals. Two hundred and six participants from Caucasian and East Asian populations were engaged in a cross-sectional study. Within a single session, they were assessed for tear film quality, visual surface characteristics, and symptoms of dry eye. In comparison to the Caucasian individuals, a greater proportion of Asian participants were found to have dry eye symptoms and meet the TFOS DEWS II dry eye diagnostic criteria. Asian participants were more likely to exhibit incomplete blinking, poor lipid layer quality, Ocular Surface Disease Index (OSDI) scores, stable tear film, lid wiper epitheliopathy, tear osmolarity, and expressed meibomian quality, all of which may have contributed to the racial tendency for the development of dry eyes [57]. As compared to Caucasian companions, demographic studies centred in East Asia have found a higher prevalence of suggestive dry eyes. Asian civilization has been identified as a persistent risk factor for the development of dry eyes in the most recent TFOS DEWS II epidemiology report. Many previous studies have also suggested that the Asian eye exhibits poor staining of the optical surface, tear film stability, and MGD resulting in greater levels of dry eye symptomology [58]. 

Nichols et al. examined 12 additional prevalence studies and discovered a combined DED pervasiveness of 17.0% in China. In contrast to 10 researches that reported DED identified by both symptoms and signs, only two out of the 12 investigations reported the occurrence of suggestive DED [59]. Due to modern lifestyles rendered worse by digital technology and the increasing ageing process, DED has become a significant public health concern in China. A thorough investigation of the incidence of DED was carried out in China in 2010; it was shown that higher latitude was a remarkable risk factor for DED by symptoms and indications [60].

According to the 2017 TFOS DEWS II epidemiology report, Asians are more likely than Caucasians to develop DED. This is supported by the inflated occurrence of DED by symptoms found in this study (31.40% in individuals aged 5-89 years) compared to that found in England (20.8% in females aged 20 and older) and the United States (15.2% in adults aged 21 and older). The study also discovered that relatively low humidity is a well-known risk factor for DED. According to this statistic, there were more Chinese individuals with DED symptoms in 2010 than there are Americans as a whole today. In modern society, visual health is very important, as prolonged visual works play a significant role in day-to-day activities. With the fast-growing aged population in China, this age-aggravated condition will expectedly be even greater in the future.

Risk factors and diagnostic tests associated with DED

On the basis of practical considerations, it has been found to be advantageous to categorize DED as "dry eye with reduced tear production (aqueous-deficient)" and "dry eye with increased evaporation of the tear film (hyperevaporative)". Only 10% of dry eye patients have a condition that is exclusively aqueous-deficient. More than 80% of instances are due to combined hyperevaporative/aqueous-deficient types and hyperevaporative disorders, which are mainly induced by MGD. New investigative techniques and corrective strategies have been developed in response to this imminent concept [61]. Risk factors for DED have been listed by the "Definition and Classification Subcommittee of the International Dry Eye Workshop" in 2007 based upon the level of evidence which has been projected in Table 1.

**Table 1:** Risk factors of DED based on the levels of evidence DED: dry eye disease; HIV/HTLV 1: human immunodeficiency virus/human T-cell lymphotropic virus Reference: [61]

Risk factors of DED having a high level of evidence	Risk factors of DED having a moderate level of evidence	Risk factors of DED having a low level of evidence
Age	Medications such as tricyclic antidepressants, selective serotonin reuptake inhibitors, diuretics, and beta-blockers	Smoking
Female sex	Diabetes mellitus	Hispanic ethnicity
Post-menopausal estrogen therapy	HIV/HTLV 1 infection	Anticholinergic drugs such as anxiolytics and antipsychotics
Antihistamines	Systemic chemotherapy	Consumption of alcohol
Collagen vascular disease	Cataract surgery with a large incision	Menopause
Corneal refractive surgery	Keratoplasty	Botulinum toxin injection
Irradiation	Isotretinoin	Acne
Hematopoietic stem cell transplantation	Low air humidity	Gout
Vitamin A deficiency	Sarcoidosis	Oral contraceptives
Hepatitis C	Ovarian dysfunction	Pregnancy
Androgen insufficiency		

**Figure 1:**
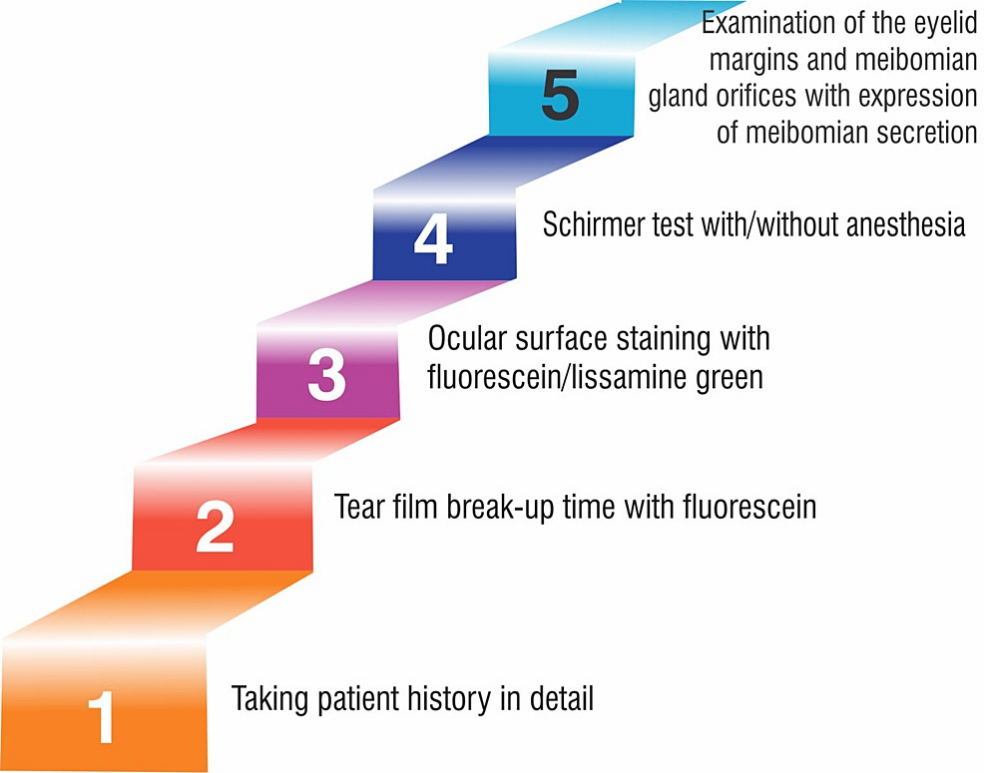
Sequence of DED diagnostic tests DED: dry eye disease Image Credit: Niyati Deo

From tear replacement alone to a coherent remedial method, dry eye care has advanced [62]. It is crucial to conduct diagnostic tests to distinguish between dry eye, infections, and allergies because these conditions might present clinically similar yet require different treatments. Dry eye may worsen if an incorrect medical diagnosis is made and anti-allergic medications are used. A thorough history of the patient, including the time, place, and diurnal variation of symptoms, medication history, systemic diseases (particularly infections such as hepatitis C, diabetes mellitus, collagen vascular disease, Graves' disease, and human immunodeficiency virus (HIV)), and workplace stress (such as visual display unit (VDU) work, dry, dusty air, and air conditioning), is necessary in addition to the sequence of dry eye tests [62]. The diagnostic tests make it simple to divide patients into two groups according to their treatment requirements: "aqueous-deficient" or "hyperevaporative". The DEWS issued diagnostic guidelines in 2007. The recommended sequence of dry eye tests according to the workshop is presented in Figure 1.

In actuality, there is still no infallible way to diagnose DED, and the only thing that can be done to cure it is to temporarily relieve symptoms and make improvements.

## Conclusions

DED is one of the most common ocular morbidities. It has been clinically subdivided into two subtypes, i.e., aqueous-deficient and hyperevaporative DED. Research has proven that DED occurs more with the increase in age for both men and women. It is also a fact that women of older age are more prone to this disease as compared to men. DED is a growing public health burden throughout the world with the increase in the number of ageing population. There is a significant relationship between dry eye and autoimmune diseases, more prevalent in women. Sex-oriented changes in the cornea of women are seen mostly in the menopausal and post-menopausal phases. Lack of overall physical health indicators in women, such as sufficient sleep, balanced nutrition, and minimal stress, is also considered a major risk factor for DED.

The occurrence of DED is much rarer in children in comparison to adults, and early detection may help to treat the underlying causes in time. It has been found that the Asian population is more symptomatic of dry eye as compared to the Caucasian people. Looking at the dearth of relevant data, it can be said that DED requires a lot of attention from researchers. Orientation and training of clinicians other than ophthalmologists are also desirable to ensure the early detection and treatment of dry eye leading to improvement in the quality of life of all the patients suffering from DED.
